# Efficacy and safety of everolimus in combination with trastuzumab and paclitaxel in Asian patients with HER2+ advanced breast cancer in BOLERO-1

**DOI:** 10.1186/s13058-017-0839-0

**Published:** 2017-04-11

**Authors:** Masakazu Toi, Zhimin Shao, Sara Hurvitz, Ling-Ming Tseng, Qingyuan Zhang, Kunwei Shen, Donggeng Liu, Jifeng Feng, Binghe Xu, Xiaojia Wang, Keun Seok Lee, Ting Ying Ng, Antonia Ridolfi, Florence Noel-baron, Francois Ringeisen, Zefei Jiang

**Affiliations:** 1 Department of Breast Surgery, Graduate School of Medicine, Kyoto University, 54 Shogoin Kawara-cho, Sakyo-ku, Kyoto 606-8507 Japan; 2 Department of Breast Surgery, Cancer Hospital of Fudan University, Shanghai, China; 3 University of California, Los Angeles (UCLA), Los Angeles, CA USA; 4 Taipei Veterans General Hospital, National Yang Ming University, Taipei, Taiwan; 5 Department of Medical Oncology, Tumor Hospital of Harbin Medical University, Harbin, China; 6 Ruijin Hospital Shanghai Jiao Tong University School of Medicine, Shanghai, China; 7 Department of Medical Oncology, Sun Yat-sen University Cancer Center, Guangzhou, China; 8 Jiangsu Cancer Hospital, 210009 Nanjing, China; 9 Department of Medical Oncology, Cancer Hospital and Institute, Chinese Academy of Medical Sciences and Peking Union Medical College, Chaoyang District, Beijing, China; 10 Department of Medical Oncology, Affiliated Zhejiang Cancer Hospital of Zhejiang Chinese Medical University Hangzhou, Hangzhou, Zhejiang Province China; 11 Center for Breast Cancer, National Cancer Center, Gyeunggi-do, South Korea; 12 Department of Clinical Oncology, Tuen Mun Hospital, Tuen Mun, Hong Kong; 13 Novartis Pharma SAS, Rueil-Malmaison, France; 14 Novartis Pharma AG, Basel, Switzerland; 15 Beijing 307 Hospital of PLA, Beijing, China

**Keywords:** Advanced breast cancer, BOLERO-1, Everolimus, HER2, Metastatic breast cancer, Asian

## Abstract

**Background:**

The current exploratory analysis was performed to evaluate the efficacy and safety of everolimus for treatment of human epidermal growth factor receptor 2-positive (HER2+) advanced breast cancer in the Asian subset of patients in the BOLERO-1 trial.

**Methods:**

Postmenopausal women with HER2+ advanced breast cancer, who had not received systemic therapy for advanced disease, were randomized 2:1 to receive everolimus or placebo, plus trastuzumab and paclitaxel. The two primary end points were investigator-assessed progression-free survival (PFS) in the full population and in the hormone receptor-negative (HR–) subpopulation. Secondary end points included assessment of the objective response rate, the clinical benefit rate, and safety.

**Results:**

In the Asian subset, median PFS was similar in the everolimus (*n* = 198) and placebo (*n* = 105) arms in the full analysis set (hazard ratio = 0.82 (95% CI 0.61–1.11)). In the HR– subpopulation, everolimus prolonged median PFS by 10.97 months vs placebo (25.46 vs 14.49 months; hazard ratio = 0.48 (95% CI 0.29–0.79)). In the everolimus arm of the Asian subset, the most common adverse events of any grade were stomatitis (62.2%), diarrhea (48.0%), rash (43.4%) and neutropenia (42.3%). Neutropenia (grade 3: 27.6%; grade 4: 4.6%) and decreased neutrophil count (grade 3: 11.2%; grade 4: 3.6%) were the most frequent grade 3/4 adverse events. Serious adverse events included pneumonia (5.1%), pneumonitis (3.1%), and interstitial lung disease (3.1%). There were three deaths (1.5%) during treatment in the everolimus arm vs none in the placebo arm.

**Conclusions:**

The efficacy and safety of everolimus plus trastuzumab and paclitaxel as first-line treatment for HER2+ advanced breast cancer in the Asian subset was consistent with that reported previously in the overall population.

**Trial registration:**

ClinicalTrials.gov, NCT00876395. Registered on 2 April 2009.

**Electronic supplementary material:**

The online version of this article (doi:10.1186/s13058-017-0839-0) contains supplementary material, which is available to authorized users.

## Background

Human epidermal growth factor receptor-2 (HER2) overexpression has been reported in approximately 20–25% of breast cancers and is historically associated with poor outcomes [1, 2]. The advent of trastuzumab, an HER2-targeted agent, has dramatically improved outcomes in patients with HER2-positive (HER2+) breast cancer [3]. Apart from trastuzumab, several other newer HER2-targeted agents (lapatinib, pertuzumab and trastuzumab emtansine (T-DM1)) have also demonstrated clinical benefit in patients with HER2+ breast cancer [4–8]. However, resistance to HER2-targeted therapies (both *de novo* and acquired) remains a clinical challenge and warrants exploration of additional treatment options, which could improve outcomes in patients with HER2+ advanced breast cancer [9, 10].

The phosphoinositide 3 kinase (PI3K)/Akt/mammalian (or mechanistic) target of rapamycin (mTOR) pathway has been implicated in the development of resistance to HER2-targeted therapies due to its crosstalk with HER2 signaling [11, 12]. In preclinical and xenograft studies, everolimus, an mTOR inhibitor, sensitized phosphatase and tensin homolog (PTEN)-null tumors to trastuzumab [13, 14]. These data suggest a potential role for the combination of everolimus and trastuzumab in the treatment of HER2+ breast cancer.

The efficacy and safety of everolimus in combination with trastuzumab plus chemotherapy has been evaluated in two phase-III clinical trials, BOLERO-1 and BOLERO-3 [15, 16]. In BOLERO-1, everolimus plus trastuzumab and paclitaxel as first-line therapy for HER2+ advanced breast cancer did not improve progression-free survival (PFS) vs trastuzumab and paclitaxel alone in the full population (median PFS 14.95 months vs 14.49 months; hazard ratio = 0.89 (95% confidence interval (CI) 0.73–1.08); *P* = 0.1166); however, there was 7.2-month improvement in PFS in the everolimus arm in the hormone receptor-negative (HR–) subpopulation (median PFS 20.27 months vs 13.08 months; hazard ratio = 0.66 (95% CI 0.48–0.91); *P* = 0.0049), which was close to, but did not cross, the protocol-specified significance threshold of *P* = 0.0044 by a small margin [16]. In BOLERO-3, the addition of everolimus to trastuzumab and vinorelbine significantly prolonged PFS compared to trastuzumab and vinorelbine alone in patients with HER2+ advanced breast cancer progressing on prior trastuzumab and taxane (median PFS 7.0 months vs 5.78 months; hazard ratio = 0.78 (95% CI 0.65–0.95)); *P* = 0.0067) [15].

Available evidence from several recent trials suggests that pharmacokinetics and pharmacodynamics of drugs may vary across ethnicities [17–22]. As a result, clinical outcomes and safety profiles in patients from one ethnic background could be different from those in another. For example, the incidence of interstitial lung disease was higher in Asian patients treated with gefitinib monotherapy compared to patients from other ethnic backgrounds [21]. Similarly, the incidence of grade 1 or 2 interstitial lung disease was higher in an Asian subset of patients in BOLERO-2, although the trial population was different (HR+, HER2– advanced breast cancer progressing on prior nonsteroidal aromatase inhibitors treated with everolimus plus exemestane) [19]. In CLEOPATRA, a phase-III trial of pertuzumab in combination with trastuzumab and docetaxel in patients with HER2+ advanced breast cancer, the overall incidence of adverse events was higher in Asian vs non-Asian patients [17]. These data highlight the importance of evaluating the clinical benefit of treatment regimens in individual ethnic subpopulations.

The current exploratory analysis of the BOLERO-1 trial evaluated the efficacy and safety of everolimus in combination with trastuzumab and paclitaxel in Asian patients with HER2+ advanced breast cancer.

## Methods

### Study design and participants

BOLERO-1 was a phase III, multicenter, international, randomized, double-blind, placebo-controlled study and has been described previously [16]. Briefly, women aged ≥18 years with HER2+ metastatic or locally recurrent invasive breast cancer were eligible [16]. Patients with no prior trastuzumab or with (neo)adjuvant trastuzumab and/or chemotherapy discontinued >12 months from randomization were eligible. Patients were not eligible for the trial if they had received systemic therapy for advanced disease, except for endocrine therapy. All patients provided written informed consent. Patients were categorized as Asian or non-Asian based on race. The study was performed in accordance with the Good Clinical Practice guidelines and the Declaration of Helsinki. The study protocol was approved by an independent ethics committee or by the institutional review boards of the participating centers.

### Procedures

BOLERO-1 procedures have been described in detail previously [16]. Briefly, patients were randomized 2:1 to receive everolimus (10 mg/day) or placebo in combination with trastuzumab and paclitaxel. Randomization was stratified by the presence of visceral metastases and prior (neo)adjuvant trastuzumab treatment. Treatment continued until disease progression, development of unacceptable toxicity, or consent withdrawal. Tumor assessments were performed 28 days prior to start of therapy (baseline assessment) and every 8 weeks after treatment initiation until disease progression based on the response evaluation criteria in solid tumors (RECIST). Efficacy end points were analyzed based on investigator assessments, and additional supportive analyses were conducted based on the central assessments. Adverse events were graded as per the National Cancer Institute Common Terminology Criteria for Adverse Events (NCI-CTCAE) version 3.0.

### Study end points

The study had two primary objectives: (1) investigator-assessed PFS in the full population and (2) investigator-assessed PFS in the HR– subpopulation. Secondary end points included assessment of the objective response rate (ORR), the clinical benefit rate (CBR), and safety.

### Statistical analyses

Statistical analysis of BOLERO-1 has been described previously [16]. For analysis of the Asian and non-Asian subsets, a non-stratified Cox regression model was used to estimate the hazard ratio for PFS and the 95% CI.

## Results

### Patient characteristics

A total of 719 patients were enrolled in the study between 10 September 2009 and 16 December 2012 [16]. Of these, 303 patients were Asian; 198 patients were randomized to the everolimus arms and 105 patients to the placebo arm. All 303 Asian patients were included in the efficacy analysis and 300 were included in the safety analysis. The baseline and disease characteristics were generally similar in the Asian and the non-Asian subset and were well-balanced between the two treatment arms. The majority of the Asian patients were of Chinese ethnicity (73.2%), followed by patients of Japanese ethnicity (13.6%). A larger proportion of Asian patients in the everolimus arm had an Eastern Cooperative Oncology Group (ECOG) performance status = 0 compared to non-Asian patients (61.1% vs 55.7%) (Table 1).

**Table 1:** Baseline and treatment characteristics of the Asian and non-Asian subsets (full analysis set)

Characteristic	Asian		Non-Asian	
	EVE + TRAS + PAC	PBO + TRAS + PAC	EVE + TRAS + PAC	PBO + TRAS + PAC
	*n* = 198	*n* = 105	*n* = 282	*n* = 134
Median age, years (range)	53.0 (23–75)	51.0 (29–82)	55.0 (26–86)	52.0 (19–77)
Race, *n* (%)				
Asian	198 (100%)	105 (100%)	0	0
Black	0	0	26 (9.2)	12 (9.0)
White	0	0	214 (75.9)	97 (72.4)
Native American	0	0	3 (1.1)	0
Other non-Asian	0	0	39 (13.8)	25 (18.7)
Ethnicity, *n* (%)				
Chinese	145 (73.2)	75 (71.4)	0	0
Indian^a^	1 (0.5%)	0	0	0
Japanese	27 (13.6)	15 (14.3)	0	0
Other Asian	25 (12.6)	15 (14.3)	0	0
Hispanic/Latino	0	0	78 (27.7)	34 (25.4)
Other non-Asian	0	0	204 (72.3)	100 (74.6)
Current disease status, *n* (%)				
Locally advanced	10 (5.1)	7 (6.7)	24 (8.5)	9 (6.7)
Metastatic	188 (94.9)	98 (93.3)	258 (91.5)	125 (93.3)
Site of metastasis, *n* (%)				
Visceral	139 (70.2)	65 (61.9)	199 (70.6)	104 (77.6)
Lung	91 (46.0)	43 (41.0)	126 (44.7)	60 (44.8)
Liver	66 (33.3)	36 (34.3)	111 (39.4)	74 (55.2)
Lung and liver	27 (13.6)	17 (16.2)	45 (16.0)	34 (25.4)
Bone	85 (42.9)	54 (51.4)	125 (44.3)	63 (47.0)
Bone only	9 (4.5)	5 (4.8)	11 (3.9)	2 (1.5)
Others	142 (71.7)	77 (73.3)	196 (69.5)	99 (73.9)
ECOG PS, *n* (%)				
0	121 (61.1)	64 (61.0)	157 (55.7)	84 (62.7)
1	77 (38.9)	41 (39.0)	125 (44.3)	50 (37.3)
ER and PgR status, *n* (%)				
ER and PgR negative (HR–)	85 (42.9)	47 (44.8)	123 (43.6)	56 (41.8)
ER and PgR negative (HR+)	112 (56.6)	57 (54.3)	159 (56.4)	78 (58.2)
Not assessable	0	1 (1.0)	0	0
Missing	1 (0.5)	0	0	0

^a^Indian subcontinent. *ECOG PS* Eastern Cooperative Oncology Group performance status, *ER* estrogen receptor, *EVE* everolimus, *HR* hormone receptor, *PAC* paclitaxel, *PBO* placebo, *PgR* progesterone receptor, *TRAS* trastuzumab

**Fig. 1:**
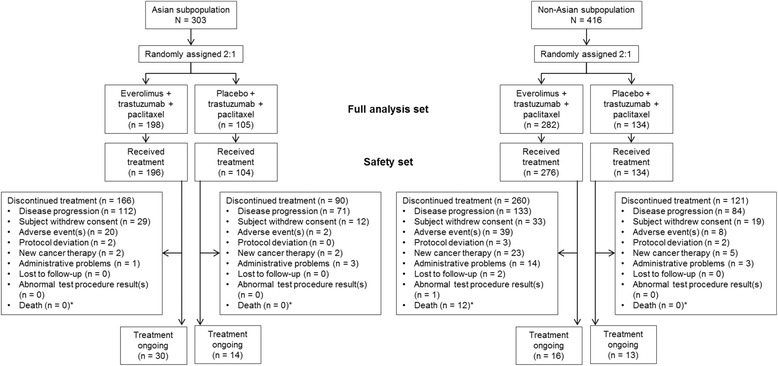
Consolidated standards of reporting trials (*CONSORT*) flowchart. *Does not include the on-treatment deaths until 28 days of follow-up

At the time of data cutoff (30 May 2014), 256 patients (84.5%) in the Asian subset discontinued treatment; 44 patients continued treatment (30 patients (15.2%) in the everolimus arm and 14 patients (13.3%) in the placebo arm). The most common reason for discontinuation was disease progression occurring in 112 patients (56.6%) in the everolimus arm and 71 patients (67.6%) in the placebo arm of the Asian subset. There was more discontinuation due to disease progression in the everolimus arm of the Asian subset (56.6%) vs the non-Asian subset (47.2%). Similarly, more Asian patients in the everolimus arm discontinued due to consent withdrawal compared to the non-Asian patients (14.6% vs 11.7%). There was less discontinuation due to adverse events in the everolimus arm in the Asian subset (10.1%) vs the non-Asian subset (13.8%) (Fig. 1).

The number of patients requiring dose reductions/interruptions was larger in the Asian subset than in the non-Asian subset, in both the everolimus and placebo arms (88.3% vs 84.4% in the everolimus arm and 79.8% vs 68.7% in the placebo arm). The most common cause of dose reduction/interruption was adverse events and this was similar in the everolimus arms of Asian and the non-Asian subset (95.4% vs 95.3%). More patients in the everolimus arm of the Asian subset experienced dose reductions/interruption due to dosing error compared to the non-Asian subset (48.0% vs 36.5%) (Additional file 1: Table S1).

### Dose intensity and exposure

The relative dose intensity of everolimus was lower in the Asian subset compared to the non-Asian subset (median 0.51 vs 0.57). The dose intensity of paclitaxel was also lower in the Asian subset compared to the non-Asian subset, in both the everolimus and placebo arms. Trastuzumab dose intensity was similar across both subsets in the everolimus and placebo arms. The duration of exposure of everolimus was longer in the Asian subset compared to the non-Asian subset (median, 42.1 weeks vs 40.0 weeks). Similarly, the duration of exposure of trastuzumab and paclitaxel was also longer in the Asian subset compared to the non-Asian subset, in both the everolimus and placebo arms. In the HR– subpopulation, the everolimus dose intensity was numerically lower in the Asian than in the non-Asian subset (median 0.50 vs 0.61), and the duration of everolimus treatment was similar in the Asian subset (45.8 weeks vs 44.7 weeks) (Table 2).

**Table 2:** Median relative dose intensities and duration of exposure in the Asian and non-Asian subsets (safety set)

	Overall population				HR– subpopulation			
	Asian		Non-Asian		Asian		Non-Asian	
	EVE + TRAS + PAC	PBO + TRAS + PAC	EVE + TRAS + PAC	PBO + TRAS + PAC	EVE + TRAS + PAC	PBO + TRAS + PAC	EVE + TRAS + PAC	PBO + TRAS + PAC
	*n* = 196	*n* = 104	*n* = 276	*n* = 134	*n* = 85	*n* = 47	*n* = 121	*n* = 56
Relative dose intensity, median (range)								
EVE	0.51 (0.03–1.00)	0.95 (0.01–1.00) (PBO)	0.57 (0.05–1.00)	0.97 (0.12–1.00) (PBO)	0.50 (0.03–1.00)	0.95 (0.13–1.00) (PBO)	0.61 (0.05–1.00)	0.96 (0.29–1.00) (PBO)
TRAS	0.96 (0.28–1.08)	0.97 (0.75–1.03)	0.96 (0.08–1.16)	0.96 (0.41–1.06)	0.98 (0.49–1.08)	0.97 (0.78–1.02)	0.97 (0.37–1.12)	0.96 (0.61–1.03)
PAC	0.65 (0.01–1.09)	0.72 (0.08–1.22)	0.72 (0.04–1.40)	0.84 (0.11–1.25)	0.65 (0.01–1.08)	0.82 (0.08–1.22)	0.73 (0.11–1.40)	0.79 (0.13–1.00)
Duration of exposure, median (range), weeks								
EVE	42.14 (0.6–241.7)	59.93 (2.0–240.7)	40.00 (1.1–233.1)	40.71 (1.1–207.4)	45.86 (0.6–210.4)	41.29 (2.1–221.1)	44.71 (1.4–233.1)	38.50 (1.1–207.4)
TRAS	58.07 (3.0–242.3)	64.07 (3.0–241.0)	45.00 (1.0–233.7)	40.43 (1.0–208.0)	58.29 (3.0–211.0)	41.00 (3.0–221.9)	49.86 (2.0–233.7)	38.50 (4.0–208.0)
PAC	36.50 (1.0–201.0)	37.14 (1.0–230.0)	27.07 (1.0–155.1)	30.00 (1.0–174.0)	35.00 (1.0–201.0)	35.00 (1.0–190.0)	28.86 (2.0–141.7)	27.00 (1.0–167.9)

*EVE* everolimus, *HR*– hormone receptor-negative, *PAC* paclitaxel, *PBO* placebo, *TRAS* trastuzumab

### Efficacy

**Fig. 2:**
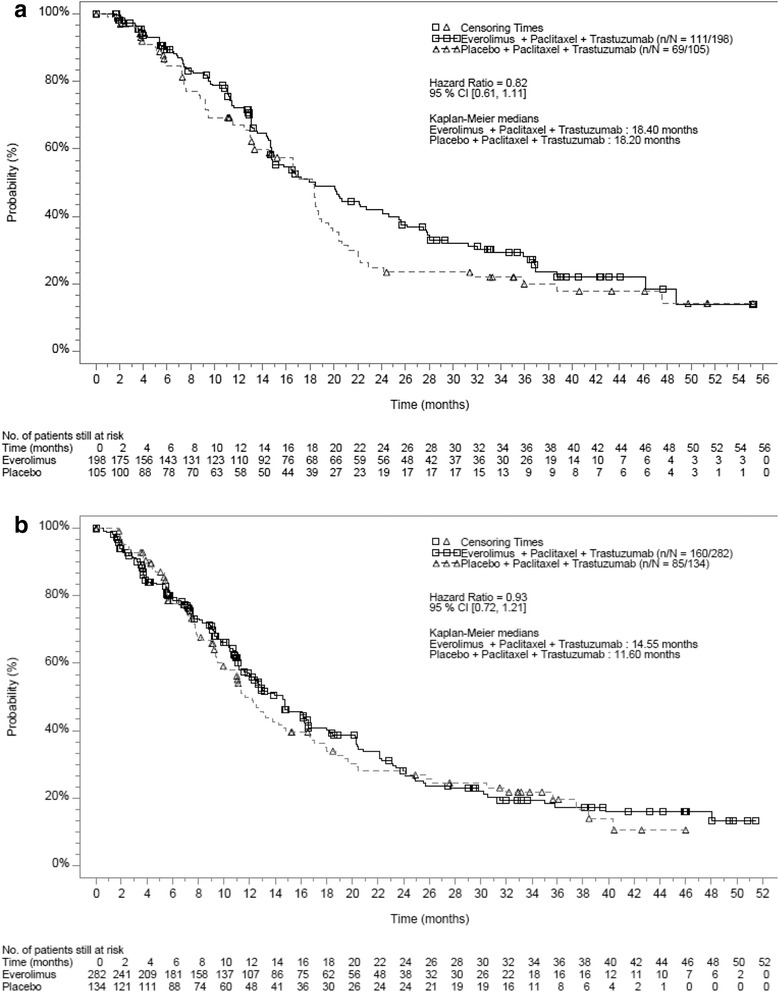
Kaplan-Meier curves for progression-free survival in the full analysis set (investigator assessment). **a** Asian subset. **b** Non-Asian subset

**Fig. 3:**
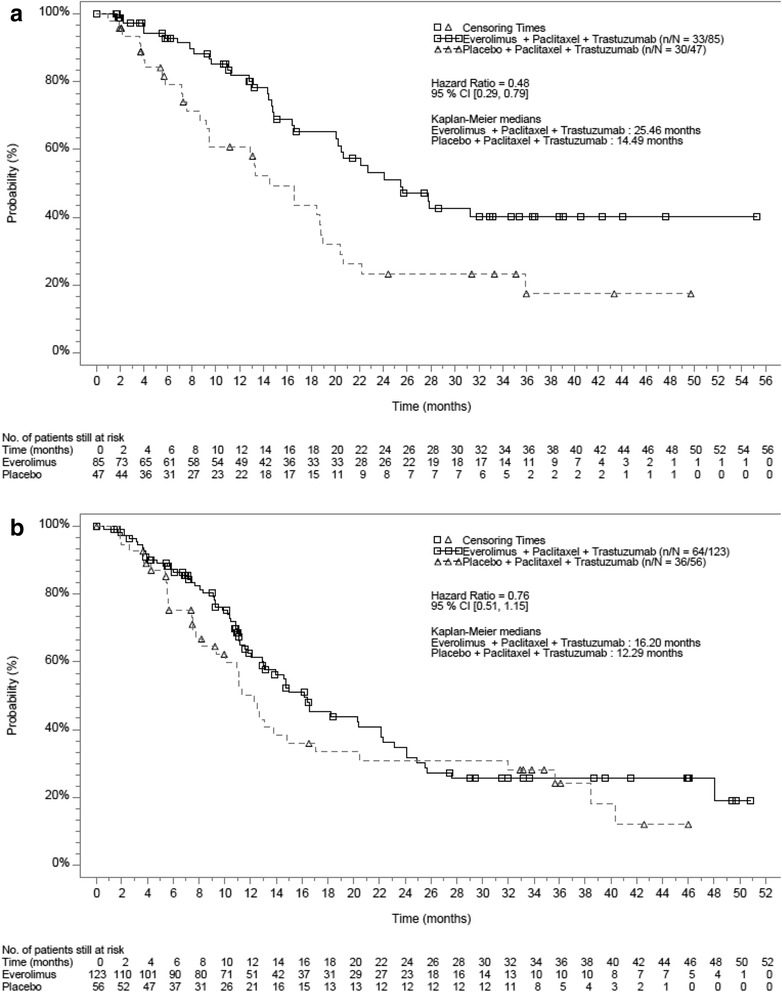
Kaplan-Meier curves for progression-free survival in the hormone receptor-negative subpopulation (investigator assessment). **a** Asian subset. **b** Non-Asian subset

In both the Asian and non-Asian subsets, the addition of everolimus to trastuzumab and paclitaxel did not improve PFS compared to placebo plus trastuzumab and paclitaxel in the full analysis set, but did prolong PFS in the respective HR– subpopulations (Figs. 2 and 3). The median PFS in the everolimus arm compared to the placebo arm in the Asian subset was 18.40 months vs 18.20 months (hazard ratio = 0.82; 95% CI 0.61–1.11) (Fig. 1). In the non-Asian subset, the median PFS in the everolimus compared to the placebo arm was 14.55 months vs 11.60 months (hazard ratio = 0.93; 95% CI 0.72–1.21) (Fig. 2).

In the HR– Asian subset, median PFS in the everolimus arm was 25.46 months compared to 14.49 months in the placebo arm (hazard ratio = 0.48; 95% CI 0.29–0.79) translating into a 10.97 months improvement in the median PFS. In the HR– subpopulation of the non-Asian subset, median PFS in the everolimus arm was 16.20 months compared to 12.29 months in the placebo arm (hazard ratio = 0.76; 95% CI 0.51–1.15) (Fig. 3).

In the Asian subset, the ORR and CBR were similar in the everolimus and placebo arms (ORR 69.2% vs 69.5%; CBR 80.8% vs 82.9%) (Additional file 1: Table S2). In the non-Asian subset, both the ORR and CBR were lower in the everolimus arm compared to the placebo arm (ORR 65.6% vs 68.7%; CBR 72.3% vs 79.9%). Both the ORR and the CBR were higher in the everolimus arm of the Asian subset than in the non-Asian subset (Additional file 1: Table S3).

**Fig. 4:**
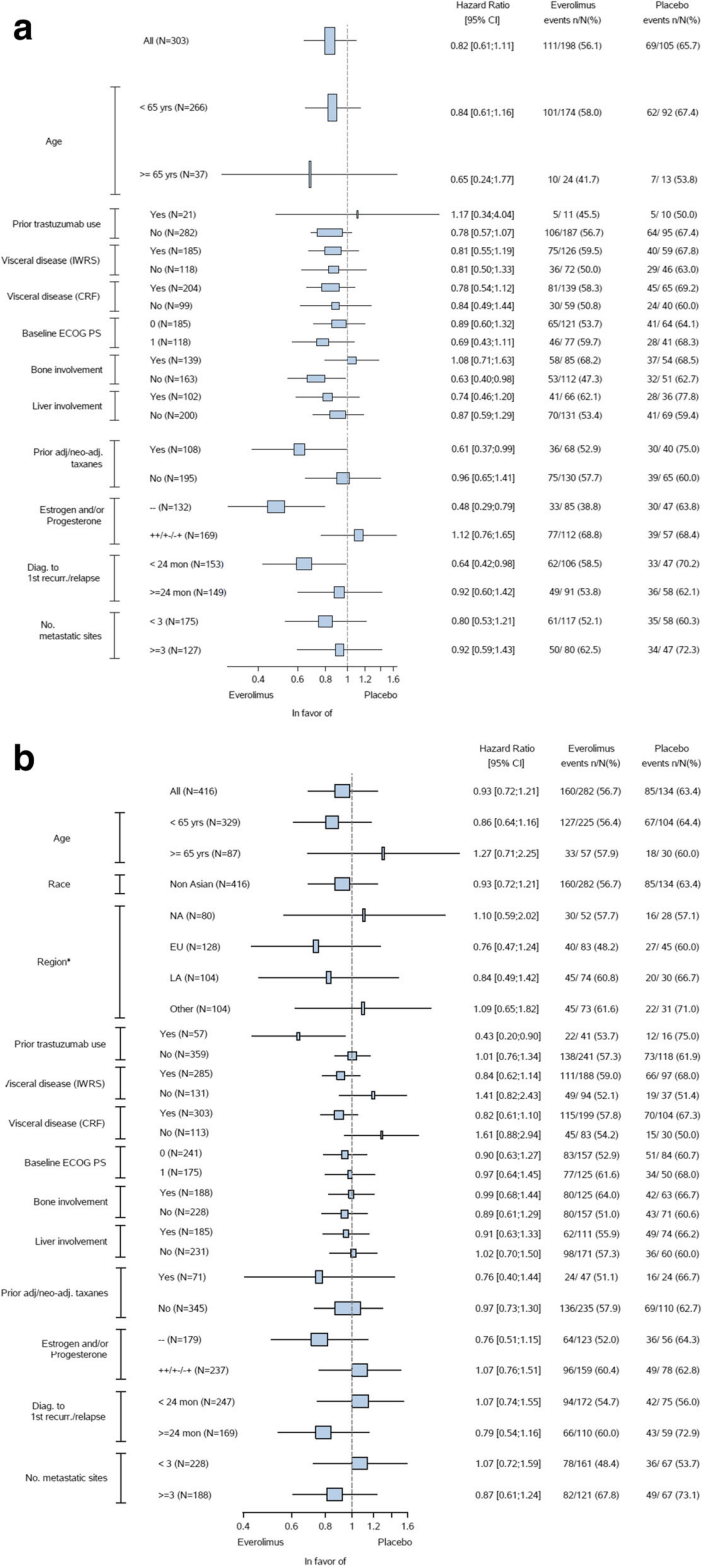
Forest plot of progression-free survival subgroup analysis (investigator assessment). **a** Asian subset. **b** Non-Asian subset. The hazard ratios were obtained using a non-stratified Cox proportional hazard model. *The majority of patients were from Asia. Due to small numbers, region is not displayed within Fig. 4. *CRF* case report form, *ECOG PS* Eastern Cooperative Oncology Group performance status, *adj* adjuvant, *diag*. diagnosis, *EU* Europe, *IWRS* interactive voice and web response system, *LA* Latin America, *NA* North America, *neo*-*adj* neoadjuvant, *recur* recurrence

PFS was also analyzed in several predefined subgroups (Fig. 4). In the Asian subset, there was a trend towards a greater PFS benefit in the everolimus arm compared to the placebo arm in patients treated with prior (neo)adjuvant taxane (n = 108) (hazard ratio = 0.61; 95% CI 0.37-.0.99), patients with HR− disease (*n* = 132) (hazard ratio = 0.48; 95% CI 0.29–0.79), patients with disease relapse <24 months after diagnosis (n = 153) (hazard ratio = 0.64; 95% CI 0.42–0.98) and in patients without bone involvement (n = 163) (hazard ratio = 0.63; 95% CI 0.40–0.98). The PFS benefit from everolimus in patients with visceral metastases (hazard ratio = 0.78; 95% CI 0.54–1.12) and in patients without visceral metastases (hazard ratio = 0.84; 95% CI 0.49–1.44) was similar to that in the overall population (Fig. 4). In general, more subgroups of Asian patients had PFS benefit with everolimus compared to the subgroups of non-Asian patients (Fig. 4).

### Safety

In the everolimus arm of the Asian subset, the most common non-hematologic adverse events of any grade were stomatitis (62.2%), diarrhea (48.0%), rash (43.4%), and alopecia (41.3%); the most common hematologic adverse events were neutropenia (42.3%), neutrophil count decrease (21.9%), and leukopenia (20.4%) (Table 3). In the everolimus arm, the incidence of pneumonia was 12.2% in the Asian subset compared to 7.6% in the non-Asian subset. The incidence of pneumonitis was 12.2% in the Asian subset compared to 19.2% in the non-Asian subset. The most frequent grade 3 and grade 4 adverse events in the everolimus arm of Asian subset were neutropenia (grade 3: 27.6%; grade 4: 4.6%) and decreased neutrophil count (grade 3: 11.2%; grade 4: 3.6%) (Table 3). In general, hematologic adverse events occurred more frequently in the Asian subset than in the non-Asian subset in both the everolimus and placebo arms. The non-hematologic adverse events that occurred more frequently in both arms of the Asian subset than in they did in the non-Asian subset included increased alanine aminotransferase, nasopharyngitis, hypoesthesia, myalgia, mouth ulceration, and neurotoxicity (Table 3). Overall, more Asian patients (34.3%) than non-Asian patients (5.9%) received granulocyte-colony stimulating factor (G-CSF). In the everolimus arm, 36.7% of Asian patients compared to 4.4% of non-Asian patients received G-CSF (Additional file 1: Table S3). The percentage of patients receiving G-CSF at each cycle of the study treatment was greater in the Asian subset compared to the non-Asian subset.

**Table 3:** Adverse events in the safety set with incidence ≥15% in the everolimus arm of he Asian or non-Asian subset

Adverse event, %	Asian						Non-Asian					
	EVE + TRAS + PAC			PBO + TRAS + PAC			EVE + TRAS + PAC			PBO + TRAS + PAC		
	*n* = 196			*n* = 104			*n* = 276			*n* = 134		
	Any grade	Grade 3	Grade 4	Any grade	Grade 3	Grade 4	Any grade	Grade 3	Grade 4	Any grade	Grade 3	Grade 4
Stomatitis	62.2	6.6	0.0	31.7	0.0	0.0	69.6	16.7	0.0	32.8	2.2	0.0
Diarrhea	48.0	6.1	0.0	40.4	3.8	0.0	62.7	11.2	0.0	51.5	4.5	0.0
Rash	43.4	1.0	0.0	24.0	1.0	0.0	38.0	0.4	0.0	17.9	0.0	0.0
Neutropenia	42.3	27.6	4.6	26.9	17.3	3.8	34.1	16.7	2.9	23.1	5.2	4.5
Alopecia	41.3	0.0	0.0	41.3	0.0	0.0	50.7	0.4	0.0	61.2	0.0	0.0
Pyrexia	39.8	0.5	0.0	26.0	1.0	0.0	38.4	2.2	0.0	26.9	1.5	0.0
Cough	38.3	0.5	0.0	35.6	0.0	0.0	41.3	0.4	0.0	30.6	1.5	0.0
Fatigue	33.2	3.1	0.0	29.8	1.9	0.0	36.6	6.2	0.0	40.3	3.0	0.0
Epistaxis	29.1	0.0	0.0	10.6	0.0	0.0	36.2	0.0	0.0	23.1	0.0	0.0
Edema peripheral	28.6	0.0	0.0	17.3	1.0	0.0	36.2	1.4	0.0	29.9	0.0	0.0
ALT increased	28.1	5.1	0.0	26.9	6.7	0.0	15.6	6.5	0.0	11.9	3.0	0.7
Nasopharyngitis	27.0	0.0	0.0	30.8	1.9	0.0	12.7	0.0	0.0	11.2	0.0	0.0
Nausea	26.0	0.5	0.0	25.0	1.9	0.0	37.3	1.1	0.0	41.8	0.0	0.0
Hypoesthesia	23.5	3.1	0.0	26.9	1.9	0.0	5.4	0.0	0.0	5.2	0.0	0.0
Mouth ulceration	23.0	1.0	0.0	10.6	0.0	0.0	5.4	0.7	0.0	1.5	0.0	0.0
Decreased appetite	22.4	1.0	0.0	16.3	0.0	0.0	23.9	1.4	0.0	13.4	0.0	0.0
Myalgia	22.4	0.0	0.0	25.0	1.0	0.0	12.0	0.0	0.0	14.2	0.0	0.0
Neutrophil count decreased	21.9	11.2	3.6	20.2	12.5	1.9	0.4	0.4	0.0	1.5	0.0	0.0
Headache	20.9	1.0	0.0	18.3	1.9	0.0	33.0	0.4	0.0	37.3	0.0	0.0
Leukopenia	20.4	8.7	0.5	14.4	5.8	1.0	11.6	4.0	0.4	6.7	3.0	0.0
Neurotoxicity	19.9	0.0	0.0	22.1	1.0	0.0	0.4	0.0	0.0	0.7	0.0	0.0
Insomnia	19.9	0.0	0.0	11.5	0.0	0.0	13.8	0.0	0.0	20.1	0.0	0.0
Vomiting	19.4	1.0	0.0	20.2	1.9	0.0	30.1	1.1	0.0	25.4	3.0	0.0
Dizziness	18.9	2.0	0.5	15.4	0.0	0.0	13.4	0.0	0.0	14.9	1.5	0.0
Weight decreased	18.9	0.5	0.0	4.8	0.0	0.0	22.5	2.2	0.0	5.2	0.0	0.0
AST increased	18.4	2.6	0.0	14.4	3.8	0.0	13.8	4.0	0.7	9.0	0.7	0.7
Dyspnea	17.3	2.0	0.0	1.0	0.0	0.0	29.3	4.3	0.7	17.2	1.5	0.0
Constipation	16.3	0.0	0.0	18.3	0.0	0.0	25.0	0.0	0.0	23.1	0.0	0.0
Peripheral sensory neuropathy	15.8	0.5	0.0	18.3	0.0	0.0	11.6	0.7	0.0	12.7	1.5	0.0
Pruritus	15.8	0.5	0.0	8.7	0.0	0.0	12.0	0.4	0.0	11.2	0.0	0.0
WBC count decreased	15.3	9.7	0.5	12.5	7.7	0.0	1.1	0.7	0.0	0.7	0.0	0.0
Oropharyngeal pain	15.3	0.0	0.0	11.5	0.0	0.0	15.9	0.0	0.0	14.2	0.0	0.0
Arthralgia	13.8	0.0	0.0	11.5	0.0	0.0	19.2	1.4	0.0	21.6	1.5	0.0
Anemia	13.3	5.6	1.0	7.7	1.9	0.0	43.5	10.5	1.4	22.4	3.0	0.0
Hypercholesterolemia	12.8	0.5	0.0	7.7	0.0	0.0	22.5	1.4	0.0	11.2	0.0	0.0
Neuropathy peripheral	12.8	0.5	0.0	15.4	3.8	0.0	40.2	6.2	0.0	31.3	5.2	0.0
Pneumonia^a^	12.2	1.5	1.0	4.8	0.0	0.0	7.6	2.9	0.0	3.7	0.0	0.0
Pneumonitis^a^	12.2	2.0	0.0	2.9	0.0	0.0	19.2	5.4	1.4	5.2	0.7	0.0
Hyperglycemia^a^	11.7	4.6	1.0	1.9	1.0	0.0	14.1	5.8	0.7	8.2	1.5	0.0
Pain in extremity	11.7	0.5	0.0	12.5	0.0	0.0	22.5	1.4	0.4	18.7	0.0	0.0
Back pain	11.2	1.0	0.0	11.5	1.0	0.0	18.1	1.1	0.0	21.6	3.0	0.0
Hypertension	11.2	2.6	0.0	11.5	1.0	0.0	17.8	2.2	0.0	10.4	2.2	0.0
Hypokalemia	10.7	5.1	0.5	2.9	2.9	0.0	17.0	7.2	2.2	3.7	0.0	0.0
Abdominal pain	10.7	1.5	0.0	9.6	0.0	0.0	18.1	0.4	0.0	14.2	0.0	0.0
Asthenia	9.2	2.0	0.0	8.7	0.0	0.0	26.8	1.4	0.0	23.9	1.5	0.0
Urinary tract infection	6.6	0.5	0.0	3.8	0.0	0.0	17.0	1.4	0.0	9.7	0.0	0.0

^a^Adverse events of special interest. *EVE* everolimus, *PAC* paclitaxel, *PBO* placebo, *TRAS* trastuzumab, *ALT* alanine aminotransferase, *AST* aspartate aminotransferase, *WBC* white blood cell

The most common adverse events leading to treatment discontinuation in the Asian subset were neurotoxicity (14.8% in the everolimus arm vs 17.3% in the placebo arm) and hypoesthesia (8.7% in the everolimus arm vs 7.7% in the placebo arm) (Additional file 1: Table S4). None of the patients in the non-Asian subset discontinued treatment due to neurotoxicity or hypoesthesia. Discontinuation of treatment due to stomatitis was more frequent in the Asian subset (5.6%) than in the non-Asian subset (2.9%), and fewer Asian patients (4.1%) discontinued treatment due to pneumonitis compared to non-Asian patients (6.9%) (Additional file 1: Table S4).

The most frequent serious adverse events in the everolimus arm of the Asian subset were pneumonia (5.1%), pneumonitis (3.1%), and interstitial lung disease (3.1%) (Additional file 1: Table S5). The most frequent serious adverse events in the everolimus arm of the non-Asian subset were pneumonitis (5.4%), stomatitis (3.6%), and dyspnea (3.6%) (Additional file 1: Table S5). The incidence of serious pneumonia (3.3%) and interstitial lung disease (0.4%) was lower in the non-Asian subset; however, the incidence of serious pneumonitis (5.4%), stomatitis, and dyspnea (3.6%) was higher in the non-Asian subset (Additional file 1: Table S5). There were three deaths on treatment (1.5%) in the everolimus arm of the Asian subset, one due to disease progression, one to pneumonia, and one to sepsis. There were 19 deaths (6.9%) in the everolimus arm of the non-Asian subset; there were no deaths on treatment in the placebo arm of the Asian subset, whereas there were 2 deaths (1.5%) in the non-Asian subset (Additional file 1: Table S6).

## Discussion

Addition of everolimus to trastuzumab and paclitaxel as first-line treatment in the advanced setting for patients with HER2+ did not have PFS benefit in the Asian subset although median PFS was prolonged in the HR– subgroup compared to the placebo arm. The safety profile of everolimus was generally similar in the Asian and non-Asian subsets, and no new safety signals were identified. These data suggest that the efficacy and safety of everolimus in combination with trastuzumab and paclitaxel as first-line treatment for HER2+ advanced breast cancer in Asian patients was consistent with that in the overall population in BOLERO-1 [16].

Some differences were noted between the Asian and non-Asian subsets in terms of efficacy, adverse event profile, and drug exposure, which could be attributed to the inherent differences in ethnic background. Among Asian, non-Asian, and the overall population, the difference in median PFS in the HR-negative subpopulation was largest in the Asian subset with a hazard ratio of 0.48 (95% CI 0.29–0.79), while in the non-Asian subset, the hazard ratio was 0.76 (95% CI 0.51–1.15). In subgroup analyses, in the Asian subpopulation, there was a trend towards a greater PFS benefit in those patients who had previously been treated with (neo)adjuvant taxane, had HR– disease, had disease relapse <24 months after diagnosis, or had no bone involvement, and who were receiving receiving everolimus. A potential explanation could be the longer duration of exposure to everolimus in the Asian subset. The lower dose intensity of everolimus in the Asian subset may have allowed patients to receive treatment for a longer duration. The duration of exposure to trastuzumab and paclitaxel was also longer in the Asian subset and could have contributed to the longer PFS.

The incidence of hematologic adverse events, notably decreased neutrophil count and leukopenia, was higher in the Asian subset (in both the everolimus and placebo arms) than in the non-Asian subset. This is consistent with the higher incidence of hematologic adverse events reported in previous studies of Asian patients with solid tumors treated with targeted therapy and/or chemotherapy [23–26] and could be attributed to the inherent ethnic differences between Asian and non-Asian subsets. Hematologic adverse events were more frequently managed using G-CSF among the Asian subset. In general, the percentage of patients receiving G-CSF decreased with each cycle of study treatment.

Some of the limitations of the present analysis include the exploratory nature and the relatively small number of patients and events, especially in the subgroup analyses. However, the pronounced PFS benefit with everolimus in the HR– subgroup of the Asian subset warrants further exploration. An evaluation of the benefit of longer duration of treatment with everolimus, with appropriate dose modification based on the ethnic background, is also warranted. Examining the underlying risk factors and molecular/physiological differences between Asian and non-Asian patients may help explain the higher incidence of hematologic adverse events reported here and in other trials.

## Conclusions

The efficacy and safety of everolimus plus trastuzumab and paclitaxel as first-line treatment for HER2+ advanced breast cancer in the Asian subset was consistent with that reported previously in the overall population. The pronounced efficacy in some subgroups and the higher incidence of hematologic adverse events warrant further investigation. Prospective trials evaluating PI3K/mTOR inhibitors are warranted, especially in Asian patients with HER2+, HR– advanced breast cancer.

## Additional file

Additional file 1: Supplementary Results. (PDF 113 kb).
Additional file 2: List of ethics committees or institutional review boards. (DOCX 15 kb)

## Abbreviations

CBR: Clinical benefit rate
CI: Confidence interval
CONSORT: Consolidated standards of reporting trials
ECOG PS: Eastern Cooperative Oncology Group performance status
EVE: Everolimus
G-CSF: Granulocyte-colony stimulating factor
HER2+: Human epidermal growth factor receptor-2 positive
HR–: Hormone receptor-negative
mTOR: mammalian (or mechanistic) target of rapamycin
NCI-CTCAE: National Cancer Institute Common Terminology Criteria for Adverse Events
ORR: Objective response rate
PAC: Paclitaxel
PBO: Placebo
PFS: Progression-free survival
PI3K: phosphoinositide 3 kinase
RECIST: Response evaluation criteria in solid tumors
T-DM1: Trastuzumab emtansine
TRAS: Trastuzumab
