# Supplementary material for: Efficacy and safety of everolimus in combination with trastuzumab and paclitaxel in Asian patients with HER2+ advanced breast cancer in BOLERO-1
Source: Breast Cancer Res. 2017 Apr 11;19:47. doi: 10.1186/s13058-017-0839-0 (PMC5387380; doi:10.1186/s13058-017-0839-0)
Supplement: Additional file 1: — Supplementary Results. (PDF 113 kb). [file 13058_2017_839_MOESM1_ESM.pdf]

Supplementary Materials

Supplementary Table 1 Dose reductions/interruptions in the Asian and non-Asian subsets (safety set)

	Asian		Non-Asian	
	EVE + TRAS + PAC n = 196	PBO + TRAS + PAC n = 104	EVE + TRAS + PAC n = 276	PBO + TRAS + PAC n = 134
Dose reductions/interruptions, n (%)*	173 (88.3)	83 (79.8)	233 (84.4)	92 (68.7)
Adverse event	165 (95.4)	59 (71.1)	222 (95.3)	69 (75.0)
Dosing error	83 (48.0)	55 (66.3)	85 (36.5)	43 (46.7)
Scheduling conflict	5 (2.9)	8 (9.6)	15 (6.4)	11 (12.0)
Concomitant medication affecting drug exposure	2 (1.2)	2 (2.4)	1 (0.4)	1 (1.1)
Lab test abnormality	-	-	1 (0.4)	0 (0.0)
Missing	6 (3.5)	3 (3.6)	6 (2.6)	2 (2.2)

*Number of patients with at least 1 dose reduction/interruption by reason
EVE everolimus; *PAC* paclitaxel; *PBO* placebo; *TRAS* trastuzumab

Supplementary Table 2 Investigator-assessed best overall response in the Asian and non-Asian subsets (full analysis set)

Response rates, n (%)	Asian		Non-Asian	
	EVE + TRAS + PAC n = 198	PBO + TRAS + PAC n = 105	EVE + TRAS + PAC n = 282	PBO + TRAS + PAC n = 134
Best overall response				
Complete response (CR)	11 (5.6)	6 (5.7)	16 (5.7)	8 (6.0)
Partial response (PR)	126 (63.6)	67 (63.8)	169 (59.9)	84 (62.7)
Stable disease (SD)	44 (22.2)	26 (24.8)	60 (21.3)	29 (21.6)
Progressive disease (PD)	3 (1.5)	4 (3.8)	13 (4.6)	7 (5.2)
Unknown	14 (7.1)	2 (1.9)	24 (8.5)	6 (4.5)
objective Response rate (ORR)*	137 (69.2 [95% CI, 62.3-75.5])	73 (69.5 [95% CI, 59.8-78.1])	185 (65.6 [95% CI, 59.7-71.1])	92 (68.7 [95% CI, 60.1-76.4])
Clinical benefit rate (CBR)†	160 (80.8 [95% CI, 74.6-86.0])	87 (82.9 [95% CI, 74.3-89.5])	204 (72.3 [95% CI, 66.7-77.5])	107 (79.9 [95% CI, 72.1-86.3])

* ORR = CR + PR
† CBR = CR + PR + SD ≥ 24 weeks
CI, confidence interval; *EVE* everolimus; *PAC* paclitaxel; *PBO* placebo; *TRAS* trastuzumab

Supplementary Table 3 Summary of G-CSF use in the Asian and non-Asian subsets (safety set)

	Asian			Non-Asian		
	EVE + TRAS + PAC	PBO + TRAS + PAC	Total (Asian)	EVE + TRAS + PAC	PBO + TRAS + PAC	Total (non-Asian)
	n = 196	n = 104	n = 300	n = 276	n = 134	n = 410
Patients receiving at least 1 G-CSF, n (%)	72 (36.73)	31 (29.81)	103 (34.33)	12 (4.35)	12 (8.96)	24 (5.85)
Number of G-CSF, median (range)	5.0 (1.0-42.0)	4.0 (1.0-55.0)	5.0 (1.0-55.0)	1.0 (1.0-32.0)	2.5 (1.0-21.0)	2.0 (1.0-32.0)

EVE everolimus; *G-CSF*; granulocyte-colony stimulating factor *PAC* paclitaxel; *PBO* placebo; *TRAS* trastuzumab

Supplementary Table 4 Adverse events leading to discontinuation in the Asian and non-Asian subsets

Adverse event, %	Asian EVE + TRAS + PAC	Asian PBO + TRAS + PAC	Non-Asian EVE + TRAS + PAC	Non-Asian PBO + TRAS + PAC
	n = 196	n = 104	n = 276	n = 134
Neurotoxicity	14.8	17.3	0.0	0.0
Hypoesthesia	8.7	7.7	0.0	0.0
Stomatitis	5.6	0.0	2.9	0.7
Peripheral sensory neuropathy	4.1	4.8	2.9	3.0
Pneumonitis	4.1	0.0	6.9	0.7
Neutropenia	3.1	1.9	2.9	0.7
Left ventricular dysfunction	3.1	0.0	2.2	2.2
Pneumonia	3.1	0.0	0.4	0.0
Mouth ulceration	2.6	0.0	0.0	0.0
Fatigue	2.0	5.8	4.0	0.7
Interstitial lung disease	2.0	0.0	0.7	0.0

EVE everolimus; *PAC* paclitaxel; *PBO* placebo; *TRAS* trastuzumab

Supplementary Table 5 Serious adverse events (incidence ≥ 2% in any arm) in the Asian and non-Asian subsets (safety set)

Adverse event, %	Asian		Non-Asian	
	EVE + TRAS + PAC	PBO + TRAS + PAC	EVE + TRAS + PAC	PBO + TRAS + PAC
	n = 196	n = 104	n = 276	n = 134
Pneumonia	5.1	0.0	3.3	0.0
Pneumonitis	3.1	0.0	5.4	0.0
Interstitial lung disease	3.1	0.0	0.4	0.0
Pyrexia	2.6	0.0	2.5	1.5
Hyperglycemia	2.6	0.0	0.4	0.0
Dizziness	2.6	0.0	0.0	0.0
Cellulitis	2.0	1.0	0.4	1.5
Dyspnea	0.5	0.0	3.6	0.7
Device related infection	0.5	1.0	3.3	0.7
Stomatitis	0.0	0.0	3.6	0.0
Infusion related reaction	0.0	0.0	0.4	2.2

EVE everolimus; *PAC* paclitaxel; *PBO* placebo; *TRAS* trastuzumab

Supplementary Table 6 On-treatment deaths in the Asian and non-Asian subsets (safety set)

Deaths, n (%)	Asian		Non-Asian	
	EVE + TRAS + PAC	PBO + TRAS + PAC	EVE + TRAS + PAC	PBO + TRAS + PAC
	n = 196	n = 104	n = 276	n = 134
Total	3 (1.5)	0	19 (6.9)	2 (1.5)
Disease progression	1 (0.5)	0	4 (1.4)	2 (1.5)
Pneumonia	1 (0.5)	0	0	0
Sepsis	1 (0.5)	0	1 (0.4)	0
Pneumonitis	0	0	3 (1.1)	0
Pulmonary embolism	0	0	2 (0.7)	0
Acute respiratory failure	0	0	1 (0.4)	0
Pulmonary edema	0	0	1 (0.4)	0
Respiratory failure	0	0	1 (0.4)	0
Pneumococcal pneumonia	0	0	1 (0.4)	0
Urosepsis	0	0	1 (0.4)	0
Cardio-respiratory arrest	0	0	1 (0.4)	0
Diabetic ketoacidosis	0	0	1 (0.4)	0
Cerebrovascular accident	0	0	1 (0.4)	0

Fall	0	0	1 (0.4)	0

EVE everolimus; *PAC* paclitaxel; *PBO* placebo; *TRAS* trastuzumab
