# Supplementary material for: Efficacy and safety of everolimus in combination with trastuzumab and paclitaxel in Asian patients with HER2+ advanced breast cancer in BOLERO-1
Source: Breast Cancer Res. 2017 Apr 11;19:47. doi: 10.1186/s13058-017-0839-0 (PMC5387380; doi:10.1186/s13058-017-0839-0)
Supplement: Additional file 2: — List of ethics committees or institutional review boards. (DOCX 15 kb) [file 13058_2017_839_MOESM2_ESM.docx]

**List of ethics committee of institutional review boards**

| **ETHICS COMMITTEE OR INSTITUTIONAL REVIEW BOARD** | **ADDRESS** |
| --- | --- |
| **Institutional Review Board of Kyoto University Hospital** | Kyoto Kyoto 606-8507, Japan |
| **Cancer Hospital of Fudan University** | Shanghai NA 200032, China |
| **Office of the Human Research Protection Program, UCLA, 11000 Kinross Avenue, Suite 102, Box 951694** | Los Angeles CA 90095-1694, USA |
| **Institutional Review Board, Taipei Veterans General Hospital, No 123** | Taoyuan County 333, Taiwan |
| **No.150, Haping Road** | Herbin Heilongjian 150000, China |
| **Ruijin Hospital Shanghai Jiao Tong Univ. School of Medicine** | Shanghai NA 200025, China |
| **Sun Yat-sen University Hospital Ethics Committee, No.561** | Guangzhou 510060, China |
| **EC of Jiangsu Cancer Hospital** | Nanjing Jiangsu   210009, China |
| **Ethics Committee of Cancer Institute and Hosptial, Chinese Academy of Medical Science, No. 17 Panjiayuan** | Beijing Chaoyang District Beijing 100021, China |
| **Ethic Committee of Zhejiang Cancer Hospital** | Hangzhou Zhejiang Province 310022, China |
| **National Cancer Center Institutional Review Board** | Goyang Gyeonggi-do 411-764, Korea |
| **New Territories West Cluster Clinical & Research Ethics Committee** | Hong Kong |
| **Ethics Committee of PLA No.307 Hospital, No.8** | Beijing Fengtai District 100071, China |
